# 

**Published:** 1853-10

**Authors:** 


Vol. VII. OCTOBER, 1853. No. 1.

THE

### DENTAL REGISTER

OF

### THE WEST.

EDITED BY

JAMES TAYLOR, M. D., D. D. S.

PUBLISHED QUARTERLY

AT $2 PER ANNUM.

AGENTS.

J. B. Dunlevy, Pittsburgh, Pa.
Drs. Spalding & Fithian, St. Louis, Mo.
Messrs. Jones, White & McCurdy, Phila. New York City, and Boston, Mass.
Dr. J. M. Brown, Cincinnati.
Solomon Brown, New York city.
John Klein, Phil. Pa.

CINCINNATI:

PRINTED BY T. WRIGHTSON, 12 WEST SECOND STREET.

1853.

